# PSP: rapid identification of orthologous coding genes under positive selection across multiple closely related prokaryotic genomes

**DOI:** 10.1186/1471-2164-14-924

**Published:** 2013-12-27

**Authors:** Fei Su, Hong-Yu Ou, Fei Tao, Hongzhi Tang, Ping Xu

**Affiliations:** 1 State Key Laboratory of Microbial Metabolism, Shanghai Jiao Tong University, Shanghai 200240, P.R. China; 2 School of Life Sciences & Biotechnology, Shanghai Jiao Tong University, Shanghai 200240, P.R. China

**Keywords:** Orthologous genes, Positive selection, Synonymous and nonsynonymous substitutions, Bacterial microevolution, *Bacillus cereus*, *Escherichia coli*

## Abstract

**Background:**

With genomic sequences of many closely related bacterial strains made available by deep sequencing, it is now possible to investigate trends in prokaryotic microevolution. Positive selection is a sub-process of microevolution, in which a particular mutation is favored, causing the allele frequency to continuously shift in one direction. Wide scanning of prokaryotic genomes has shown that positive selection at the molecular level is much more frequent than expected. Genes with significant positive selection may play key roles in bacterial adaption to different environmental pressures. However, selection pressure analyses are computationally intensive and awkward to configure.

**Results:**

Here we describe an open access web server, which is designated as PSP (Positive Selection analysis for Prokaryotic genomes) for performing evolutionary analysis on orthologous coding genes, specially designed for rapid comparison of dozens of closely related prokaryotic genomes. Remarkably, PSP facilitates functional exploration at the multiple levels by assignments and enrichments of KO, GO or COG terms. To illustrate this user-friendly tool, we analyzed *Escherichia coli* and *Bacillus cereus* genomes and found that several genes, which play key roles in human infection and antibiotic resistance, show significant evidence of positive selection. PSP is freely available to all users without any login requirement at: http://db-mml.sjtu.edu.cn/PSP/.

**Conclusions:**

PSP ultimately allows researchers to do genome-scale analysis for evolutionary selection across multiple prokaryotic genomes rapidly and easily, and identify the genes undergoing positive selection, which may play key roles in the interactions of host-pathogen and/or environmental adaptation.

## Background

With the next-generation sequencing data “tsunami” in our midst, sets of closely related prokaryotic genomes suitable for comparative evolutionary studies have been available [1]. To date, well established cases of gene selection have been rare [2]. Big data mining of bacterial genomes has shown that positive selection is more widespread at the molecular level than expected under a restrictive interpretation of the neutral theory [3]. Genome-wide molecular selection analyses, designed to assess selection pressure across the entire genomes of different strains, have attempted to address the role of gene selection in the process of microevolution [4,5], especially in host-pathogen interactions, and metabolic adaptation to a changing environment (stress, antibiotic). Positive selection studies on model bacteria, such as the species *Escherichia coli*[4,6] and *Listeria*[7], or the genera *Streptococcus*[8,9] and *Campylobacter*[10] have revealed that positive selection is an essential part of natural selection to fix advantageous mutations, and improves the adaptability of bacteria in a wide range of environmental conditions.

A number of methods have been proposed for detecting positive selection in DNA or protein sequences [2]. The most common approach is to integrate evolutionary features into codon-based models, and to use probability-based theory to estimate the ratio (*ω*) of nonsynonymous (*d*_*N*_) and synonymous (*d*_*S*_) substitutions, such as implemented in the PAML [11] and FitModel [12]. Estimating the ratio *ω* gives a measure of selective pressure, indicating neutral evolution (*ω* = 1), purifying selection (*ω* < 1) and positive selection (*ω* > 1). In the model of neutral evolution, the likelihood that a nonsynonymous mutation would go to fixation is the same as that for a synonymous mutation. Purifying selection can result in stabilizing selection through the purging of deleterious variations that arise. Positive selection pressure serves to maintain a given set of adaptive traits that aids in survival.

Several nice tools are currently available, such as IDEA [13], JCoDA [14] and WSPMaker [15]. However, they are not set up specifically to examine prokaryotic genomes, and they exhibit two major deficiencies: (i) the evolution selection analysis could be difficult to configure on a local computer for most biologists who are not familiar with phylogenetic or evolutionary theory, and (ii) excessively long computing times for analyzing several genomes at once are prohibitive. In this study, we present an open access web server called PSP (Positive Selection analysis for Prokaryotic genomes) to identify orthologous coding genes under positive selection across closely related prokaryotic genomes. It provides several core functions for in-depth analysis of evolutionary selection: retrieving the orthologous groups, generating codon-delimited and un-gapped alignments, removing recombination, building phylogenetic trees, and estimating *ω* under different models used by PAML/FitModel. Remarkably, PSP is able to facilitate efficient exploration of the identified orthologous genes at the metabolic pathway level by assignments and enrichments of KO (KEGG Orthology), GO (Gene Ontology) or COG (Clusters of Orthologous Groups) terms. Results are presented in a user-friendly web interface, which provides an efficient visualization of positive selection pressure on each orthologous groups.

## Implementation

**Figure 1:**
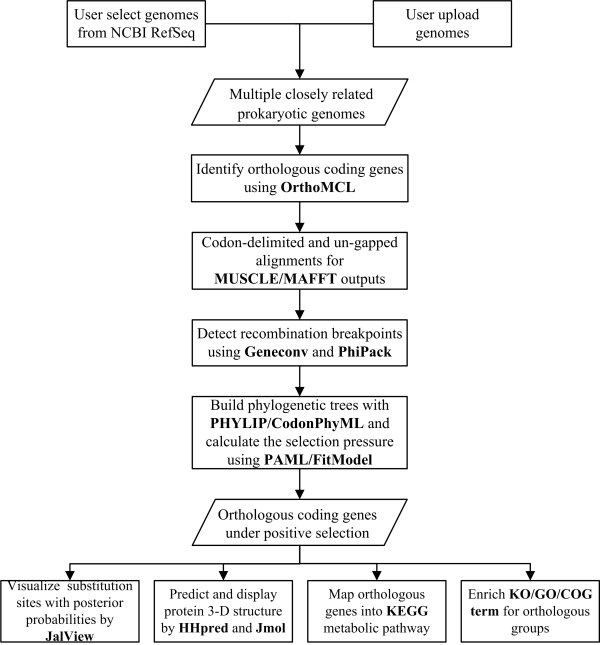
The strategy used by PSP to calculate prokaryotic selection pressure across multiple closely related genomes.

The PSP server first employs the BLAST-based orthologous genes assignment tools to build orthologous groups across multiple prokaryotic genomes being compared. Then it uses the codon-based strategy, similar to that of Petersen *et al.*[6], for identification of orthologous coding genes under positive selection. Finally, PSP facilitates functional exploration of the identified orthologous genes under positive selection by KEGG pathway mapping and enrichment of KO, GO or COG terms. Here, we briefly describe PSP as an integrated framework of PAML, FitModel and several in-house programs as follows (Figure 1).

### Rapid identification of orthologous groups across multiple prokaryotic genomes

The identification of orthologs is an important problem in the field of phylogenetic analyses. Basically, there are three types of relationships between orthologous genes, one-to-one, one-to-many and many-to-many [16]. In the study of Adrian *et al.*[17], OrthoMCL shows a balanced performance, such as the accuracy, the number of genomes analyzed and usability of the web-interface. Therefore, PSP integrates OrthoMCL to quickly identify the many-to-many orthologous relationship. PSP allows users to select or upload complete sequences and annotation details of closely related genomes for comparison. Users can also simultaneously upload thousands of annotated protein-coding genes as Multi-Fasta formatted files. With the default settings, OrthoMCL recognizes co-ortholog relationships with a BLASTp *E*-value cutoff of 1e-5 and a minimum of 50% coverage. Then PSP performs homolog grouping using the Markov Cluster algorithm with an inflation value of 1.5. Interestingly, PSP runs on a high-performance server and can accept up to thirty comparator bacterial genomes simultaneously. Moreover, one-to-one orthologous relationship of genes could be identified using reciprocal BLAST best hit, which is the first and most widely used method for automatically establishing orthologous relationships [18].

### Optimization of multiple sequence alignment for automated phylogenetic analysis

The protein-coding genes of the individual orthologous groups can be aligned by using MUSCLE or MAFFT. In connection with the automated phylogenetic analysis, PSP improves the coding sequence alignments by using the two following processes: (i) generation of codon-delimited alignments with *ad hoc* Perl scripts; (ii) maximization of the un-gapped alignment area by using MaxAlign to remove non-homologous sequences [19].

### Removal of gene recombination off orthologous groups

Recombination (or gene conversion) is 10–50 times more likely to cause changes in nucleotide sequence than mutation [20]. To eliminate the influence of horizontal relationships in the positive selection detection, PSP is able to identify recombination signals among the aligned nucleotide sequences of orthologous groups. PSP performs a statistical test to identify recombination breakpoints by using GeneConv [21]. It pre-defines the *g*-scale parameter as 1, which allows mismatches within a recombining fragment, and the inner fragment *P*-values with 10,000 random permutations. In addition, PSP also detects recombination with three other statistical tests implemented with the PhiPack package with default settings, including pairwise homoplasy index (PHI), Max χ^2^ and neighbor similarity score (NSS) [22]. Finally, if a recombination signal is detected to be significant by all four tests (*P*-value < 0.05 in each method), the alignment would be splitted into two or more fragments.

### Detection of orthologous genes under positive selection

Phylogenetic trees are built by the PHYLIP using maximum parsimony or neighbor-joining method. The trees are also able to be generated with Markov codon models by using CodonPhyML [23]. The evolutionary selection is subsequently implemented in the program PAML or FitModel. Only orthologous groups with enough data (at least 4 protein-coding genes) are used as input to detect positive selection, due to the poor quality of Bayes predictions based on small samples [24]. Because the lack of any methods to deal with alignment gaps properly in both programs, a cutoff of the percentages of sequence have data in PSP is used to filter the alignments column by column. In the PSP server, PAML uses three evolutionary models proposed by Yang *et al.*[25] (Additional file 1: Table S1): site model, strain-specific branch model and strain-specific site-branch model. In the strain-specific analysis, the branches of selected target strains are specified and referred to as “foreground branches” and the rest as “background branches”, which is a powerful tool to detect the selection pressure during the process of environmental adaptation [4]. The *in silico* detection of evolutionary selection is computationally intensive, particularly using Bayes empirical Bayes to determine posterior probabilities (PP). PSP, which runs on a high-performance server, is able to rapidly calculate the *d*_*N*_/*d*_*S*_ ratios and screen orthologous coding genes under positive selection. Similarly, PSP also can apply switching Markov modulated codon models as implemented in the program FitModel to orthologous coding genes to accurately estimate the strength of selection. To most biologists who are not familiar with phylogenetic or evolutionary theory, PSP pre-defines the evolutionary models as described in Additional file 1: Table S1. PSP is also very flexible to set most key parameters in the PAML/FitModel and run strain-specific analysis freely for the evolutionary researchers. For each pair hypothesis, nested models are calculated by comparing the difference in log likelihood values to a χ^2^ statistic (LRT) for the detection of positive selection. If there is significant evidence for positive selection of any fragment, the orthologous genes, from which the fragment was separated, are suggested to be under positive selection. Notably, PSP also provides a user-friendly visualization tool for performing evolutionary analysis on orthologous coding genes. The embedded Java applet JalView [26] reports the PP values for all sites, which is helpful for users to determine nucleotide substitution at synonymous and nonsynonymous sites within protein-coding regions. Three-dimensional structural models of the protein of interest are predicted and displayed by HHpred [27] and Jmol [28]. Additionally, Primer3Plus [29] is integrated to facilitate design of PCR primers to assay orthologous genes based on similar selection among a panel of strains isolated from the same habitat.

### Functional investigation at metabolic pathway level

To explore functions of the identified orthologous coding genes under positive selection, PSP performs KO mapping and GO/COG classification. First, to assign the KO terms, PSP uses the level of sequence identity and ratio of matching length to query length cut-off obtained from BLASTp. For each query against the locally installed KEGG gene database, the simple *H*_*a*_-value homology score [30] is calculated as follows: *H*_*a*_ = *i* × (*l*_*m*_/*l*_*q*_), where *i* is the level of identity between protein sequences in the region with the highest Bit score expressed as a ratio between 0 and 1, *l*_*m*_ the length of the highest scoring matching sequence (including gaps), and *l*_*q*_ the query length. In this study, the *H*_*a*_-value cutoff of 0.7 was used to assign KO. PSP then enriches metabolic pathways with genes under positive selection by tracing back the hierarchical KO levels. The user can select one or more KEGG-archived closely related genomes (up to 20) as references. PSP calculates the *P-*value of each pathway category by using a hyper-geometric distribution method [31]. In the same way, PSP provides enrichment analysis for COG functional terms based on RPSBLAST searches against the local CDD database, and GO slims analysis based on GOA database.

In the PSP pipeline, a large number of hypotheses are considered which could result in a high rate of Type-1 error even for a relatively stringent *P*-value cutoff. To reduce Type-1 errors, PSP corrects the obtained *P*-values using the Q-value [32] to produce a *Q-*value at level of FDR < 0.2.

## Results and discussion

The PSP tool is applicable to a wide range of prokaryotic species. In this study, we applied PSP to do genome-wide positive selection analyses in two cases (*Escherichia coli* and *Bacillus cereus*) both to benchmark PSP and to illustrate its accuracy and usefulness for the exploration of data.

### Positive selection analysis of *Escherichia coli*

**Figure 2:**
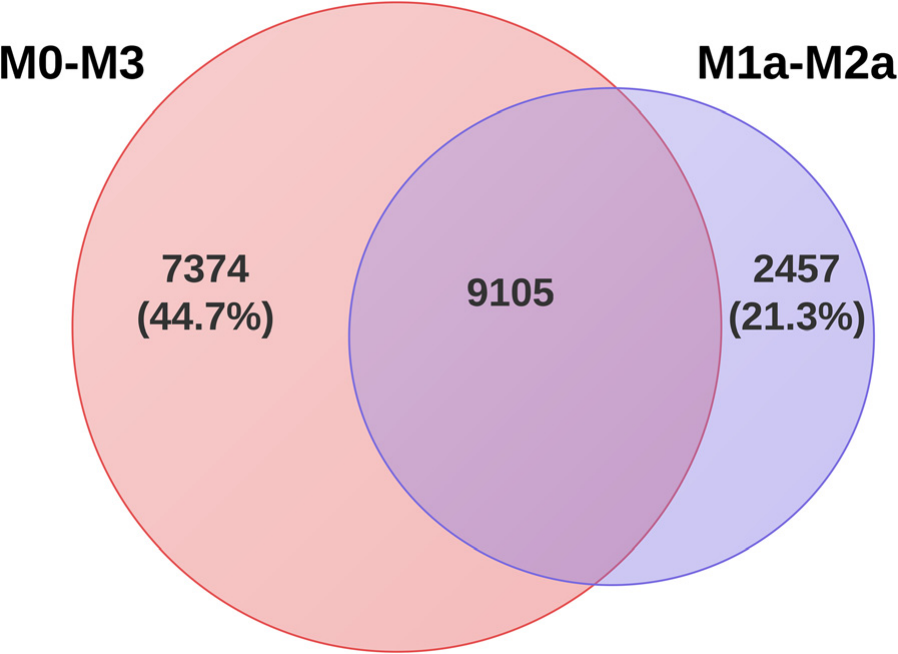
**Venn graph of the sites under positive selection from different models.** The pink represents the sites from Model M0-M3, while the purple represents those from Model M1a-M2a.

We did a genome-wide positive selection analysis in four *E. coli* and two *Shigella flexneri* genomes (Additional file 1: Table S2), which were used by Petersen *et al.*[6]. The results of selection analysis in *E. coli* by Petersen *et al.* differ from those by Chen *et al.*[4] in several important ways, which only four genes were identified in both studies. To clarify the positive selection pressure of *E. coli*, we used six different models (M0, M1a, M2a, M3, M2a + S1, M2a + S2) to test for positive selection using PAML and FitModel. Additional file 1: Table S3 shows the *P*-values from LRTs obtained from four comparisons (M0-M3, M1a-M2a, M2a-M2a + S1 and M2a + S1-M2a + S2) examined in this case. According to the idea of Yang (See detail from PAML FAQ), different evolutionary models always produce various parameter estimates and possibly different lists of site under positive selection. However, the sites under positive selection with posterior probabilities (PP > 99%) in one model are more likely to detect under different models. The tests of M2a-M2a + S1 and M2a + S1-M2a + S2 could not access the points, which evolved under positive selection yet. Therefore, we compared the sites with PP > 99% across the remaining models (11,562 sites from model M2a and 16,479 from M3), in which about 9,105 (78.7%) sites from M2a shared with the result of model M3 (55.2%, Figure 2). In the previous studies, the M1a-M2a comparison also appears to be much more robust [33]. To ensure accuracy of the result, we used the results of M1a-M2a tests for further analysis. This analysis took about twenty hours to run the whole pipeline with M1a-M2a tests. During the analysis, we have identified 5,393 orthologous groups, among which 3,908 groups within at least 4 genes were analyzed by PSP (Table 1). Moreover, ninety-seven genes were detected to have significant signals for recombination by using both GenConv and PhiPack. GeneConv could also predict breakpoints where recombination has occurred. These breakpoints define fragments, which have different evolutionary histories due to recombination. Then, these orthologous genes were splitted into different fragments. In addition, about 117 genes were excluded due to MaxAlign, which was used to maximize the number of amino acid symbols that are present in gap-free columns. At last, we detected strong signals of positive selection pressure on 28 genes in *E. coli* K12, of which 13 genes were in the “core” groups (orthologous genes present in all genomes), while 15 genes were in the “dispensable” groups (orthologous genes present in two or more genomes but not all). However, there are still some differences between the results of Petersen and ours. This is primarily because Petersen focused on their studies specific to *E. coli* K12 and disregarded the orthologous groups that contain no K12 gene. In contrast, the methods used here identified selection pressure that acts in either “core” groups or “dispensable” groups. We identified 13 additional “dispensable” orthologous groups under positive selection. What’s more, the different strategy of tests for recombination also affected the result of positive selection analysis. To estimate the accuracy of PSP’s, we compared the *d*_*S*_ value with previous studies, that the *d*_*S*_ value is fairly independent across different datasets [13]. We calculated a median *d*_*S*_ of 88.7 × 10^-3^ among *E. coli* strains with the M0 model. The value we obtained is consistent with previous estimates of 27.0 - 51.3 × 10^-3^ (*P*-value < 0.001, Wilcoxon test) [34].

**Table 1:** Results of positive selection scanning during the analysis pipeline

**Results**	**This research**	**Petersen *et al.***	**Chen *et al.***
Num. of orthologous groups	5,393	-	-
Num. of orthologous (groups size > = 4)	3,908	3,757	3,470
Num. of genes removed by MaxAlign	117	-	-
Method for detecting recombination	GenConv & PhiPack	HyPhy	GeneConv & Reticulate
Num. of recombination	97	239	443
Evolutionary model	M1a-M2a	M1a-M2a & M7-M8	M1a-M2a & Branch-Site Model
Num. of genes under positive selection in *E. coli* K12	28	23	29

According to the result of Petersan, we found 6 genes that were under positive selection in both studies. Meanwhile, our approach resulted in the identification of 13 additional genes (excluding the genes involved in phage), including several that are very important in human infection and antibiotic resistance (Table 2). The gene *fli*C, which shows strong evidence for positive selection, encodes flagellin and is one of the most important virulence factors of Uropathogenic *Escherichia coli* (UPEC) [35]. Motility causes ascension of UPEC from bladder into kidney and helps UPEC to efficiently colonize the urinary tract. The gene *dac*C, first identified in *B. subtilis* genomes, encodes a putative 491-residue protein with homology to low-molecular-weight penicillin-binding proteins (PBPs) [36]. Expression of *dac*C in *E. coli* showed that this gene encodes an approximately 59-kDa membrane-associated PBP, which is highly toxic when overexpressed. The gene *eut*A, which encodes the reactivating factor for ethanolamine utilization, is under the strong selection pressure. And recent researches imply that the utilization of ethanolamine provides a useful source of carbon/nitrogen that promotes successful colonization of the intestine [37]. Studies have also demonstrated that mutation in the *nfs*A is associated with nitrofuran resistance, because it encodes cellular reductase that can reduce members of the nitrofuran family [38]. *hch*A and *ydd*V both are the key genes involved in resistance of a wide range of stress, such as heat and antibiotic [39,40]. The other genes, *yeb*N (conserved inner membrane protein) and *yge*R (lipoprotein), have no clear function. But properties predicted from their sequence suggest that they encode the proteins locating on the membrane surface. They may possibly serve as receptors for phages or antibiotics.

**Table 2:** **List of additional genes under positive selection across the six *Escherichia coli *** **genomes using the M1a-M2a test**

**Gene**	***P*-value**^**a**^	**KO**^**b**^	**COG**	**Pathway**	**Enrichment**^**c**^	**Function**
*ala*S	0.000	K01872	J	ko00970	0.3094	Alanyl-tRNA synthetase
*cob*U	0.046	K02231	H	ko00860	0.6536	Bifunctional cobinamide kinase and cobinamide phosphate guanylyltransferase
*dac*C	0.024	K07258	M	ko00550	0.6806	D-alanyl-D-alanine carboxypeptidase
*eut*A	0.000	K04019	E	ko00564	0.6025	Reactivating factor for ethanolamine ammonia lyase
*fli*C	0.000	K02406	N	ko02020	0.1939	Flagellar filament structural protein
*hch*A	0.000	K05523	R	-	-	Hsp31 molecular chaperone
*nfs*A	0.000	K07734	K	-	-	Nitroreductase A
*pgl*	0.000	K07404	G	ko00030	0.3210	6-phosphogluconolactonase
*yad*B	0.000	K01894	J	-	-	Glutamyl-Q tRNA synthetase
*yeb*N	0.000	-	S	-	-	Conserved inner membrane protein
*ydd*V	0.000	K13069	T	-	-	Predicted diguanylate cyclase
*yfd*F	0.041	-	-	-	-	Hypothetical protein
*ygf*I	0.013	-	K	-	-	Putative DNA-binding transcriptional regulator
*yge*R	0.000	K12943	M	-	-	Lipoprotein

^a^ The *P*-value shown is the smallest one among different fragments if there is a strong signal of recombination.

^b^ “-” indicates that no functional term was assigned.

^c^ The “enrichment” represents *P*-value of hypergeometric distribution for each KEGG pathway.

### Genes under positive selection in *Bacillus cereus* genomes

**Figure 3:**
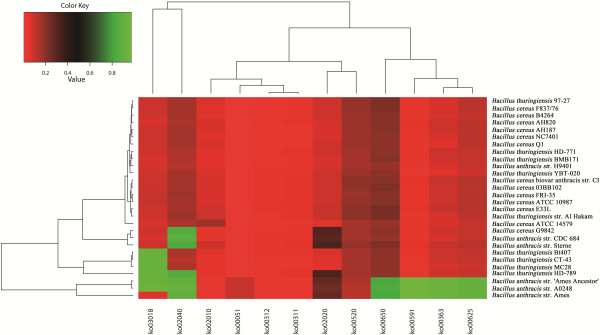
**PSP-provided heat-map showing KO (KEGG Orthology) enrichments for orthologous coding genes under positive selection across 28 completely sequenced *Bacillus *genomes.** The color scale was calculated as *P*-value of hypergeometric distribution. The x axes represents pathway of the KEGG database. The y axes represents the *Bacillus* strains analyzed in this research.

A genome-wide molecular selection scanning using PSP was also done for *Bacillus cereus* group (twenty-eight completely sequenced strains of *B. cereus*, *B. thuringiensis* and *B. anthracis* listed in the Additional file 1: Table S4). PSP compared all the 159,473 annotated genes in the 28 *Bacillus* genomes with maximum likelihood and strain-specific branch-site model using *B. anthracis* as “foreground branches” in about two hundred hours. Totally, thirty-two groups showed evidences for positive selection after correcting for multiple tests, of which 14 groups were the “core” genes while 18 groups were the “dispensable”. As in previous studies [4,6], we found that proteins that typically undergo positive selection are involved in interaction with the phages or infections (Table 3). For example, alkyl hydroperoxide reductase is an enzyme for protection strains from peroxide-induced stress and plays key roles not only in colonization but also in potential virulence [41]. In addition, a number of genes also show evidence for positive selection with the connection to antibiotic resistance, mass transport system and transcriptional regulation (Figure 3).

**Table 3:** **Genes that show evidences for positive selection in *B. anthracis *** **group**

**Gene**^**a**^	***P*-value**^**b**^	**KO**^**c**^	**COG**^**c**^	**Function**
*amp*C	0.000	K01467	V	β-lactamase
*ubi*E	0.000	-	H	SAM-dependent methyltransferase
*rlu*A	0.000	-	J	Ribosomal large subunit pseudouridylate synthase D
*glp*F	0.000	-	G	Glycerol uptake facilitator protein
*flg*L	0.000	-	N	Flagellin
*pbp*2	0.000	-	M	Penicillin-binding protein
*pai*B	0.000	K07734	K	Protease synthase and sporulation negative regulatory protein
*ara*J	0.000	-	G	ABC transporter
*bae*S	0.000	-	T	Sensor histidine kinase
*afu*B	0.000	K02011	P	Iron (III) transport system permease protein
*ptr*2	0.000	-	E	Multidrug resistance protein
*ahp*F	0.000	K03387	O	Alkyl hydroperoxide reductase, subunit F
COG3708	0.000	-	S	Transcriptional regulator, AraC family
*gcd*1	0.000	K00966	MJ	GMPP; mannose-1-phosphate guanylyltransferase
COG4653	0.000	-	R	Putative prophage LambdaBa04, major capsid protein
*rsu*A	0.000	K06183	J	RNA pseudouridine synthase family protein
*pur*R	0.000	-	K	Sugar-binding transcriptional regulator, LacI family
*cwl*A	0.000	K01447	M	N-acetylmuramoyl-L-alanine amidase
*ntr*B	0.000	K00936	T	Sporulation kinase
COG1079	0.000	-	R	ABC transporter, permease
*rnj*	0.000	K12574	R	RNA-metabolising metallo-beta-lactamase
COG3127	0.000	-	Q	ABC transporter, permease
COG1979	0.000	K00100	C	Alcohol dehydrogenase
*fep*D	0.000	K02015	P	Iron complex transport system permease protein
*nup*C	0.000	K03317	F	Nucleoside permease NupC

More details are available at http://202.120.45.186/~webserver/kaks/detail.php?jobId=UrK5YPUZE1.

^a^ Only the genes, which have clear function, were listed in this table.

^b^ The *P*-value shown is the smallest one among different fragments if there is a strong signal of recombination.

^c^ “-” indicates that no functional term was assigned.

**Figure 4:**
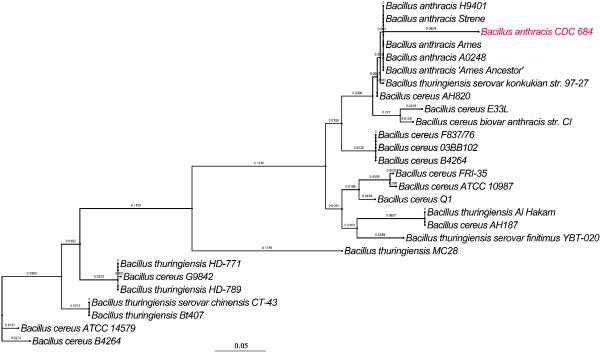
**Maximum likelihood tree of *amp *C genes from all analyzed *Bacillus *strains.** The *amp*C gene from *B. anthracis* CDC 684 was marked with red and removed by MaxAlign during the positive selection scanning. A scale bar for the genetic distance is shown at the bottom.

**Figure 5:**
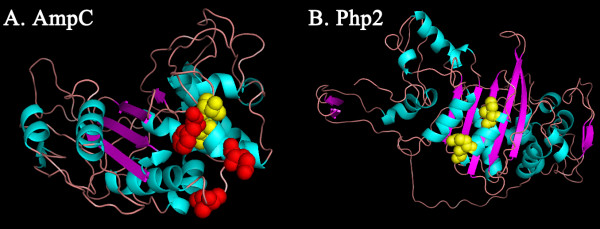
**Three-dimensional structures of AmpC and Pbp2 proteins. A)** Three-dimensional structure of β-lactamase (AmpC). **B)** Three-dimensional structure of penicillin-binding protein (Pbp2). Sites that show evidences for positive selection (PP > 99%) are depicted as red spheres. And sites (PP > 95%) are depicted as yellow spheres.

Antibiotic is well recognized to impose strong selective pressure. Especially the proteins on the surface of cell, which are more likely to be exposed, are preferential target for antibiotic-resistance-related selective pressure. In this case, we identified a β-lactamase with strong positive selection (*amp*C, *P*-value < 0.001), which could provide resistance to β-lactam antibiotics like penicillins and cephamycins. We built the phylogenetic tree of *amp*C genes from all analyzed *Bacillus* strains (Figure 4), in which all from *B. anthracis* are clustered together. However, the gene from *B. anthracis* str. CDC 684 shows significant difference from the others and was removed by MaxAlign. Susceptibility to β-lactam-containing compounds is a common trait of *B. anthracis*, because gene expression of β-lactamase is usually not sufficient to provide resistance to the agents [42]. Therefore, β-lactam agents have been used worldwide to treat anthrax, which would speed up the evolution of genes and aid bacteria populations in becoming resistant to antibiotics. And at the same time, *php*2, which is a type of PBPs, is under strong positive selection. The strong selection pressure is attributable to the process of “arms race” between antibiotic and pathogen. With the protein structures of AmpC and Php2, the majority of sites predicted to be under positive selection are surface exposed (Figure 5). Positive selection in the Php2 seems to be associated with selection to avoid recognition by the antibiotic.

Mass transport system is another effective way for microorganism to resist antibiotic, although it is probable that they may have other natural physiological functions [43]. There are seven genes involved in the mass transport system, which shows the significant enrichment compared to the references (*P*-value = 0.0395 in the strain Ames Ancestor). The *ara*J gene was regarded as nonessential membrane protein of unknown function. Recently, it is believed to belong to a large class of multidrug resistance translocators and in particular to the major facilitator superfamily [44]. The orthologous genes of *ptr*2, which encode a peptide transporter, present in all *B. cereus* genomes and are regarded as a multidrug resistance protein. In this research, we found it is under strong positive selection and may have important roles to extrude drugs. Iron acquisition genes are also important contributors to *B. anthracis*, while iron limitation is a component of host defense against infection [45]. The gene *fep*D, which shows strong evidence for positive selection, is a virulence-associated gene and involved in iron-chelating ABC uptake systems [46]. The gene *afu*B was reported to differentially express upon treatment with antibiotic, and is also very important for iron transporter [47]. Porins are also important in interaction with the host immune system and could work as receptors for phages or antibiotics [6]. Aquaglyceroporin *glp*F selectively conducts the passage of small hydrophilic across the inner membrane of *B. cereus*[48]*.* The function of remaining two putative ABC transporters is still unknown, but one of them (COG3127) was predicted to involve in lysophospholipase L1 biosynthesis, which may be a good potential target for new antibiotics.

We also identified two transcriptional repressors (*pur*R and *ara*C) and a two-component system (*ntr*B-*ntr*C), which show strong evidences for positive selection. Autoregulation of *pur*R controls the expression of many genes involved in purine biosynthetic pathway in *B. subtilis*[49]. *ntr*B, a member of the *ntr*B-*ntr*C two-component system, encodes the signal-transducing kinase/phosphatase nitrogen regulator, on the regulated phosphatase activity involved in nitrogen regulation. These genes are known to have multiple functions in different ways, which are likely that the positive selection from the interaction of host-pathogen simultaneously improve the adaptation of microorganisms by acting on the versatile proteins, such as metabolic adaptation.

## Conclusions

The PSP web server, which integrates a wide variety of useful analytical and functional tools, has been developed to rapidly identify orthologous coding genes under positive selection across up to thirty user-selected or user-supplied prokaryotic genomes. Hosted by a high-performance server and with easy navigation and flexible input options, we present it as a quick and comprehensive genome microevolution tool for biologists. Remarkably, PSP excludes the effect of gene recombination and incorporates functional investigation at the metabolic pathway level. In the future, we plan to improve the computing power of PSP markedly with Paralleled PAML. The upgraded version will be also able to map the positive selection sites to three-dimensional structures of proteins. We propose that a tool such as PSP will support genome-scale analysis for evolutionary selection, aimed at defining genomic biomarkers of evolutionary lineage, phenotype, pathotype, environmental adaptation and/or disease-association of diverse bacterial species.

## Availability and requirements

Project name: PSP:Positive Selection analysis for Prokaryotic genomes.

Project home page: http://db-mml.sjtu.edu.cn/PSP/, or http://202.120.45.186/~webserver/kaks/.

Operating system(s): CentOS.

Programming language: PHP 5.3+, JavaScript, HTML5, MySQL, PERL, R.

License: PSP is available free of charge to academic and non-profit institutions.

Any restrictions to use by non-academics: Please contact authors for commercial use.

## Competing interests

The authors declare that they have no competing interests.

## Authors’ contributions

FS designed and implemented the PSP project. FS and HO drafted the paper. HT and FT contributed important ideas and advices. PX supervised the project. All authors read and approved the final manuscript.

## Supplementary Material

Additional file 1: Table S1 Evolutionary models provided by PSP. **Table S2.** Six *Escherichia coli* genomes used in case one. **Table S3.***P*-values of 4-pair comparisons of genes which found under positive selection by Petersen *et al.***Table S4.** Twenty-eight completely sequenced *Bacillus cereus* genomes under analysis in case two. Click here for file
