# Supplementary material for: PSP: rapid identification of orthologous coding genes under positive selection across multiple closely related prokaryotic genomes
Source: BMC Genomics. 2013 Dec 27;14:924. doi: 10.1186/1471-2164-14-924 (PMC3882776; doi:10.1186/1471-2164-14-924)
Supplement: Additional file 1: Table S1 — Evolutionary models provided by PSP. Table S2. Six Escherichia coli genomes used in case one. Table S3.P-values of 4-pair comparisons of genes which found under positive selection by Petersen et al.Table S4. Twenty-eight completely sequenced Bacillus cereus genomes under analysis in case two. [file 1471-2164-14-924-S1.doc]

### ADDITIONAL FILE DATA

**Table S1.** Evolutionary models provided by PSP.

**Table S2.** Six *Escherichia coli* genomes used in case one.

**Table S3.** *P*-values of 4-pair comparisons of genes which found under positive selection by Petersen *et al.*

**Table S4.** Twenty-eight completely sequenced *Bacillus cereus* genomes under analysis in case two.

**Table S1. PAML-used evolutionary models provided by PSP**

| **Model** | **Description** | **Pre-defined parameter by PSP** |
| --- | --- | --- |
| **Site model** |  |  |
| M0 | Sites on all branches have the same *ω* | One site class, *ω* |
| M1a (neutral) | Sites on all branches nearly neutral | Two site classes, *ω*_0_ < 1 and *ω*_1_ = 1 |
| M2a (selection) | Sites on all branches positive selection | Three site classes, *ω*_0_ < 1, *ω*_1_ = 1 and *ω*_2_ > 1 |
| M3 | *ω* vary among all sites discretely | Three site classes, *ω*_0_, *ω*_1_and *ω*_2_ |
| M7 (beta) | *ω* vary among all sites according to a β distribution | The *ω* ratio for a codon randomly taken from the β distribution |
| M8 (beta & *ω*) | An extra category of positively selected sites | The *ω* ratio assumed to be beta-distributed, the proportion of codons with *ω* > 1, and *ω*_s_, the value of *ω* in these sites |
| M8a (beta & *ω_s_* = 1) | 50:50 mixture of point mass 0 and χ_1_^2^ | The *ω* ratio assumed to be beta-distributed and *ω_s_* = 1 |
| S1 | Switching substitution categories under the constraint that α = β = 1 | - |
| S2 | Switching substitution categories under a potentially biased pattern | - |
| **Strain-specific branch model** | |  |
| Model one | One *ω* ratio for all branches | Model = 0, all branches have the same *ω* ratio |
| Model two | Two ω ratios for all branches: one ratio for the foreground lineage; the other for the rest lineages | Model = 2, there are two ω ratios, one for the foreground lineages and the other for the rest lineages. The foreground linages are only from the selected target species. |
| **Strain-specific branch-site model** | |  |
| Model A | Sites on the foreground lineages allowed to differ | The model assumes four site classes. Site class 0 is conserved throughout the tree, with 0 < *ω_0_* < 1 estimated. Site class 1 is evolving neutrally throughout the tree with *ω_1_* = 1. Site classes 2a and 2b are conserved or neutral on the background branches, but become under positive selection on the foreground branches with *ω_2_* > 1, estimated from the data. The foreground linages are only from the selected target species. |
| Model A1 | Sites on the foreground lineages fixed at *ω* = 1 | Fix *ω*_2_ = 1 |

**Table S2. Six *Escherichia coli* genomes used in case one**

| **Strain** | **RefSeq Accession No.** |
| --- | --- |
| *Escherichia coli* K12 str MG1655 | NC_000913 |
| *Escherichia coli* CFT073 | NC_004431 |
| *Escherichia coli* O157:H7 Sakai | NC_002695 |
| *Escherichia coli* O157:H7 EDL933 | NC_002655 |
| *Shigella flexneri* 2457T | NC_004741 |
| *Shigella flexneri* 301 | NC_004337 |

**Table S3. *p*-values of 4-pair comparisons of genes which found under positive selection by Lise *et al.***

| **Gene** | **M0-M3** | **M1a-M2a** | **M2a-M2a+S1** | **M2a+S1-M2a+S2** | **Function** |
| --- | --- | --- | --- | --- | --- |
| *fhu*A | 0.000 | 0.104 | 1.000 | 1.000 | Outer membrance protein receptor for ferrichrome |
| *eae*H | 1.000 | 1.000 | 1.000 | 1.000 | Attaching and effacing protein |
| *nmp*C | 0.000 | 1.000 | 0.000 | 1.000 | Outer membrance porin protein, pathogenesis factor |
| *ubi*F | 0.001 | 1.000 | 1.000 | 1.000 | 2-Octoprenyl-3-methyl-6-methoxy-1,4-benzoquinon hydroxylase |
| *omp*F | 0.006 | 0.949 | 1.000 | 1.000 | Outer membrance protein 1a |
| *omp*A | 0.000 | 0.500 | 0.427 | 1.000 | Outer membrance protein 3a |
| *ycg*V | 1.000 | 1.000 | 1.000 | 1.000 | Putative adhesion and penetration protein |
| *yci*D | 0.000 | 1.000 | 0.833 | 1.000 | Putative outer membrane protein |
| *rzp*R^a^ | - | - | - | - | Putative Rac prophage endopeptidase |
| *tra*8_2 | 0.000 | 0.000 | 1.000 | 1.000 | IS30 transposase |
| *tra*8_3 | 0.000 | 0.000 | 1.000 | 1.000 | IS30 transposase |
| *ydd*K | 0.000 | 0.000 | 1.000 | 1.000 | Putative glycoprotein |
| *pqq*L | 0.000 | 0.000 | 1.000 | 1.000 | Putative zinc protease |
| *noh*A^a^ | - | - | - | - | DNA packaging protein UN1 homolog from lambdoid prophage Qin |
| *pur*R | 0.000 | 0.0723 | 0.005 | 1.000 | Transcriptional repressor for pur regulon |
| *yee*U | 0.000 | 0.060 | 0.586 | 1.000 | the YeeV-YeeU toxin-antitoxin pair |
| *yee*V | 0.003 | 1.000 | 0.194 | 1.000 | the YeeV-YeeU toxin-antitoxin pair |
| *mdt*C | 0.000 | 1.000 | 0.091 | 1.000 | multidrug efflux transport system |
| *omp*C | 0.000 | 0.000 | 0.000 | 1.000 | Outer membrane protein 1b |
| *ins*A_6 | 1.000 | 1.000 | 1.000 | 1.000 | IS1 protein InsA |
| *rfa*C | 0.000 | 1.000 | 1.000 | 1.000 | Heptosyl transferase I |
| *wec*D^a^ | - | - | - | - | dTDP-fucosamine acetyltransferase |
| *lam*B | 0.000 | 1.000 | 1.000 | 1.000 | Phage lambda receptor protein; maltose high-affinity receptor |

More details are available at http://202.120.45.186/~webserver/kaks/detail.php?jobId=QWMUv1JlBX (M0-M3), http://202.120.45.186/~webserver/kaks/detail.php?jobId=fIaPhViw95 (M1a-M2a), http://202.120.45.186/~webserver/kaks/detail.php?jobId= c3HL6BiHGh (M2a-M2a+S1) and http://202.120.45.186/~webserver/kaks/detail.php?jobId=5tmQNVpTC2 (M2a+S1-M2a+S2).

^a^ The gene was filtered by the size of orthologous group ( < 4).

**Table S4. Twenty-eight completely sequenced *Bacillus cereus* genomes under analysis in case two ^a^**

| **Strain** | **Accession** | **Source** | **Note ^b^** |
| --- | --- | --- | --- |
| *B. cereus* FRI-35 | NC_018491 | - | Anthrax |
| *B. cereus* biovar anthracis CI | NC_014335 | Human | Anthrax |
| *B. cereus* 03BB102 | NC_012472 | Human | Pneumonia |
| *B. cereus* B4264 | NC_011725 | Human | Pneumonia |
| *B. cereus* AH820 | NC_011771 | Human | Periodontal disease |
| *B. cereus* F837/76 | NC_016779 | Human | Gastroenteritis |
| *B. cereus* AH187 | NC_011654 | Human | Food poisoning |
| *B. cereus* ATCC 10987 | NC_005707 | Cheese | Food poisoning |
| *B. cereus* ATCC 14579 | NC_004722 | Human | Food poisoning |
| *B. cereus* E33L | NC_007105 | Zebra | Food poisoning |
| *B. cereus* NC7401 | NC_016771 | - | Food poisoning |
| *B. cereus* G9842 | NC_011772 | Human | Food poisoning |
| *B. cereus* Q1 | NC_011969 | Soil | Industrial |
| *B. anthracis* str. 'Ames Ancestor' | NC_007530 | Human | Anthrax |
| *B. anthracis* str. A0248 | NC_012659 | Human | Anthrax |
| B. anthracis str. Ames | NC_003997 | Soil | Non-Pathogen |
| *B. anthracis* str. CDC 684 | NC_012581 | Human | Anthrax |
| *B. anthracis* str. H9401 | NC_017729 | Human | Anthrax |
| B. anthracis str. Sterne | NC_005945 | Soil | Non-Pathogen |
| *B. thuringiensis* BMB171 | NC_014171 | - | - |
| *B. thuringiensis* Bt407 | NC_018877 | Soil/Plant | Sotto disease |
| *B. thuringiensis* HD-771 | NC_018500 | - | - |
| *B. thuringiensis* HD-789 | NC_018508 | - | - |
| *B. thuringiensis* MC28 | NC_018693 | - | - |
| *B. thuringiensis* serovar chinensis CT-43 | NC_017208 | - | - |
| *B. thuringiensis* serovar finitimus YBT-020 | NC_017200 | Soil/Plant | Sotto disease |
| *B. thuringiensis* serovar konkukian str. 97-27 | NC_005957 | Soil | Sotto disease |
| *B. thuringiensis* str. AI Hakam | NC_008600 | Soil | Sotto disease |

^a^ See Table S3 for the PSP-identified orthologous groups. More details are available at http://202.120.45.186/~webserver/kaks/detail.php?jobId= UrK5YPUZE1.

^b^ The disease data were taken from PATRIC (http://patricbrc.vbi.vt.edu/portal/portal/patric/Home).
